# Sharing an Open Stimulation System for Auditory EEG Experiments Using Python, Raspberry Pi, and HifiBerry

**DOI:** 10.1523/ENEURO.0524-20.2021

**Published:** 2021-08-24

**Authors:** Alexandra Corneyllie, Fabien Perrin, Lizette Heine

**Affiliations:** Auditory Cognition and Psychoacoustics Team, Lyon Neuroscience Research Center, CNRS-UMR 5292, Institut National de la Santé et de la Recherche Médicale U1028, Université Claude Bernard Lyon 1, 69675 Lyon, France

**Keywords:** auditory stimulation, stimulus synchronization, EEG, low-cost I/O device, timing accuracy, OpenHardware, OpenScience, Raspberry Pi

## Abstract

In auditory behavioral and EEG experiments, the variability of stimulation solutions, for both software and hardware, adds unnecessary technical constraints. Currently, there is no easy to use, inexpensive, and shareable solution that could improve collaborations and data comparisons across different sites and contexts. This article outlines a system composed by a Raspberry Pi coupled with Python programming and associated with a HifiBerry sound card. We compare its sound performances with those of a wide variety of materials and configurations. This solution achieves the high timing accuracy and sound quality important in auditory cognition experiments, while being simple to use and open source. The present system shows high performances and results along with excellent feedback from users. It is inexpensive, easy to build, share, and improve on. Working with such low-cost, powerful, and collaborative hardware and software tools allows people to create their own specific, adapted, and shareable system that can be standardized across different collaborative sites, while being extremely simple and robust in use.

## Significance Statement

The present system shows the sustainability of using low-cost creation tools to solve recurrent constraints in neuroscience experiments. It contributes to the Open Science movement from hardware making to Open Source software and includes all the content necessary for the readers to build and develop their own system.

## Introduction

Collaboration between centers is not always easy with regard to hardware and software compatibility. Currently, software for stimulation protocols in behavioral and electrophysiological assessments (e.g., having subjects hear a specific auditory stimuli) can be expensive and proprietary, and often requires technical support (whether open source or not). Even when using fixed software, there is still a wide variability because of computer hardware and installations. These are likely to vary from one laboratory to another because of differences in operating system, drivers, or updates between studies. Even small differences in terms of hardware or software might have repercussions, which are not always tested and reported in studies and can lead to important sources of error impacting the replication in studies (Plant and Quinlan, 2013). For example, hardware and software variations are likely to lead to deviations in the presentation and synchronization timing (i.e., onset and jitter). This will therefore influence analysis and results, particularly in research areas in which a strong temporal accuracy is required, as for example in time-locked behavior or brain activity analysis (e.g., reaction time, evoked related potentials, phase coherence). The temporal accuracy of the analysis is directly dependent on the temporal accuracy of the stimulation system. It is necessary, for example, to have a jitter of <1 ms to be able to interpret the cortical responses with a temporal precision of ∼10 ms.

A wide range of software is available to present stimulations to human participants in behavioral and electrophysiological (including EEG) experiments, such as famous programs like Presentation (https://www.neurobs.com/), OpenSesame (Mathôt et al., 2012), Eprime (Taylor and Marsh, 2017), Psychopy (Peirce, 2007), and Psychtoolbox (Borgo et al., 2012), the latter in MATLAB. All of the software allows for use of experimental control and management, resulting in good time performances for experiments. They vary both in terms of specialization of specific functionality and research area. However, all of the software is still dependent on computer hardware, drivers, and operating systems (OS), resulting in variability of configuration settings and installations (Plant et al., 2004; Garaizar et al., 2014).

In addition, the trigger information from the stimulation computer (or other devices, such as response boxes) to the acquisition computer (e.g., in EEG recording) is generally sent through the parallel port (PP; also named DB25 or LPT [line printing terminal] port, from the **“**D-Sub” connector family), which is reliable, easy to use, and has excellent timing accuracy (Stewart, 2006; Voss et al., 2007). Most conventional EEG systems have a PP as entry for triggers (e.g., BrainAmp, BrainProducts; EGI, Philips) or use similar single-bit technologies (37 pin D-Sub connector, BioSemi). However, the PP is nowadays mostly replaced by other ports such as the universal serial bus (USB; Canto et al., 2011). The lack of these PPs in newer hardware and associated support in recent OS and programming environments is a major problem as this often leads in practice to the use of old material without warranty or the newest updates, or inappropriate systems [e.g., PCMCIA (Personal Computer Memory Card International Association) parallel port adapters as cited in Psychology software tools information (https://support.pstnet.com/hc/en-us/articles/229359707-INFO-Recommended-Parallel-Port-Adapters-for-Machines-without-a-Parallel-Port-18031-); and risk of chipset, BIOS, or OS incompatibilities with extension card, as cited in the Brain Product press release (https://pressrelease.brainproducts.com/triggerbox-tips/)].

Another possible solution is to add an external PP hard PCI (peripheral component interconnect) card, which could be plugged to the motherboard of tower computers, but this does not solve the drivers and material variability. These solutions could lead to heavy technical work at every update and the need to maintain old versions of protocols, which is not efficient in terms of fragmenting the maintenance efforts for such systems.

In addition to these issues, the current stimulation systems are often not easily transportable and shareable. There is thus a strong need to use more standardized materials that are accessible (i.e., inexpensive, open source, easy to use, and easy to adapt for a specific context) as well as durable (e.g., choices in updating, tracking library versions, cross-platform), powerful, portable, shareable, and, in the best scenario, sustained by a community of users.

Several studies have made great strides in this direction and have shown that advances in computer portability through open-source technology can contribute to (neuro-)scientific studies. For example, Kuziek et al. (2017) showed that a Raspberry Pi2 can be used to present auditory (beeps) oddball paradigms. Another example of a Raspberry Pi use in multisite neuroscientific studies results from multisensory studies on rodent behavior (Buscher et al., 2020). Others have shown that an Arduino could be easily used for steady-state visual evoked potential (Mouli et al., 2015). However, these systems have been developed for very simple protocols and materials (beeps) and could be improved both in timing accuracy and audio quality. The need to develop a more generalized protocol player, a highly sound and timing efficient, shareable, and transportable system is part of a general move in research toward more open science. More and more possibilities are being created for sharing of data, analysis [e.g., Open Science Framework (https://osf.io)], and protocols such as the OpenBehavior Project (White et al., 2019).

Here we show that a Raspberry Pi 3, together with a HiFiBerry (additional sound card to Raspberry Pi), allows for high stereo sound quality and good timing performances (in terms of latency and jitter) while being powerful, easy to use, inexpensive, and in an open source ecosystem (software and hardware). We developed a Python library allowing the presentation of any auditory material for behavioral or electrophysiological studies, from simple to sophisticated paradigms. We also provided specifics for a state-of-the-art stimulation box for auditory experimental protocols. This box includes a parallel port for EEG synchronization of triggers, a Raspberry Pi 3, HiFiBerry, and battery. This portable system box can be used in fundamental and translational research settings. It can significantly reduce variation between studies and/or centers as well as simplify the setup of this kind of protocol. It has been developed in a multisite collaboration involving laboratory and clinical environments, thanks to the CogniComa project, and the box is actually used with four protocols in Lyon and Toulouse (e.g., mismatch negativity protocols, language and music protocols, math protocols).

Ultimately, we designed this stimulation system as a shareable project and encouraged its dissemination, use, and future improvements.

## Materials and Methods

We designed the system as follows: a “playframe” (simple organized comma-separated file document) carries the information about the protocol (ordered sounds to play and their associated timing and triggers). A Python library allows the playing process (either on a PC or in combination with the proposed hardware) and a new stimulation box based on a Raspberry Pi as hardware to propose a possible open standard.

### Playframe

**Figure 1.**
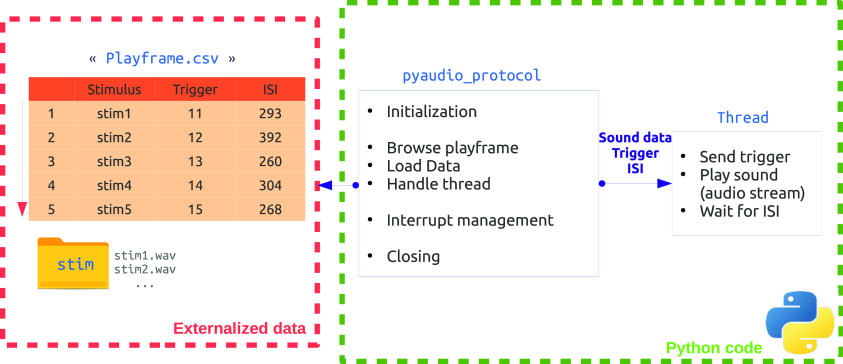
Playframe and software process. Left, An example of a playframe file consisting of columns for stimuli, associated triggers, and ISI (i.e., the time between stimuli). Right, The PyAudioProtocol core, which uses the playframe to send information to a dedicated thread. This thread plays the sound while sending the associated triggers.

The playframe is the input of the reading process and consists of a simple table with the following three columns: the name of the .wav file to be played, the associated trigger value, and the associated timing (Fig. 1). The latter consists of time in milliseconds from the end of the current sound to the beginning of the next sound [i.e., the interstimulus interval (ISI)]. This table should be named “Playframe,” saved as a “.csv” or “.xls” file, and constitutes a protocol playlist in a pandas (https://pandas.pydata.org/) dataframe style. It is protocol specific and generated independently of the player. An example of playframe generation with pandas is available in https://github.com/PyOpenProto/PyOpenProto/tree/e-neuro2021/examples/playframe_generation_example. The playframe could then be set on an external USB key, with relative audio stimuli and plugged in to the stimulation box to play the corresponding protocol. This separation between protocol-specific content (USB key) and general reading purpose (stimulation box) makes it possible to switch very easily between different protocols (or different subjects orders) without any change in the playing process, while maintaining the exact same system efficiency across protocols (or subjects). Associated auditory stimuli should be in standard stereo .wav audio format with a 44,100 Hz sampling rate in a “stim” dedicated folder.

### Software

The dissociation of protocol content and playing process allows the player implementation to focus on general core accuracy in timing and sound quality while bypassing specific ordering complexity (Fig. 1). The use of such external session list imports is generally provided in most presentation software.

The Python programming language for the playing process was the obvious coding solution. It allows for interoperability (coupled with a wide range of available powerful libraries), readability, efficiency, and is associated with a large collaborative community (Muller et al., 2015). The *Python in neurosciences* ecosystem provides an excellent unifying solution addressing the needs of both scientists and engineers, from data acquisition to treatment and analysis.

We designed a Python library named PyAudioProtocol, which is a part of the more general project ‘PyOpenProto’ available on GitHub (https://github.com/PyOpenProto/PyOpenProto/tree/e-neuro2021) PyAudioProtocol relates to pure audio stimuli presentation coupled with parallel port triggering, subject to strong constraints in sound quality and timing accuracy. This library hosts two versions for auditory stimulus presentation. One version is intended for classical computers (‘core_gui.py’ file), which expects the presence of a parallel port to send the triggers to the EEG acquisition system. The second version allows the use of the Raspberry Pi3-based hardware described in the next section (‘core_rpi_nogui.py’ file). This version simulates a parallel output via GPIO (general purpose input/output). Both versions were tested, compared, and used, as described in the Results section and follow the same simple code architecture described below.

The PyaudioProtocol reads the playframe (Fig. 1) and loads the data, after which it initializes a thread using the python-sounddevice (https://python-sounddevice.readthedocs.io) module, which provides the bindings to the PortAudio (http://www.portaudio.com/) library to benefit from precise audio-streaming management. At the same time, a trigger is sent using pyparralel (https://github.com/pyserial/pyparallel) or Rpi.GPIO (https://pypi.org/project/RPi.GPIO/) from PC or Raspberry Pi3 versions, respectively. The next trigger and sound will be sent to the output device after the given ISI. In parallel to this, interruption management allows the protocol to be stopped at any given time. Finally, PyaudioProtocol allows the correct initialization and closing down of material and programming objects used.

### Hardware

The hardware can be a standard computer with a parallel port (internal or external, PCI card recommended in this case), to be used with the computer code version available on GitHub (https://github.com/PyOpenProto/PyOpenProto/blob/e-neuro2021/pyaudio_protocol/core_gui.py).

A standardized, portable, and accessible system using a Raspberry Pi is also proposed. The Raspberry Pi3 B+ board was used, which natively has a low sound quality and a variable trigger-tone latency, as previously described by Kuziek et al. (2017). To overcome those limitations, the HiFiBerry DAC+ pro [digital analog converter (DAC)] was used as an additional sound card. It has a dedicated 192 kHz/24 bit high-quality Burr Brown DAC coupled to an ultra-low-noise voltage regulator for best sound quality and a dual-domain clock circuit to produce low-jitter performance. [THD+N (total harmonic distortion plus noise): −92 dB, signal-to-noise ratio = 112 dB]. This card, with its low-jitter clock generator and optimal audio performances, ensures access to most of the Raspberry Pi GPIO that could be used for other purposes.

The Raspberry Pi3 itself uses the Raspbian OS (https://www.raspberrypi.org/software/operating-systems/). This open source system is installed on a classic secure digital card and is easily replicable. A small number of specific configurations was adapted. Precisely, we included the HiFiBerry audio output, activated SSH (secure shell protocol for cryptographic networking) and automatic logging. In addition, we used an ARM I2C (microprocessor interintegrated circuit) interface and allowed for an automatic run of PyAudioProtocol starting script when the system switches on. A ready-to-use operating system with all those configurations is available for download on https://osf.io/3muqk/.

**Figure 2.**
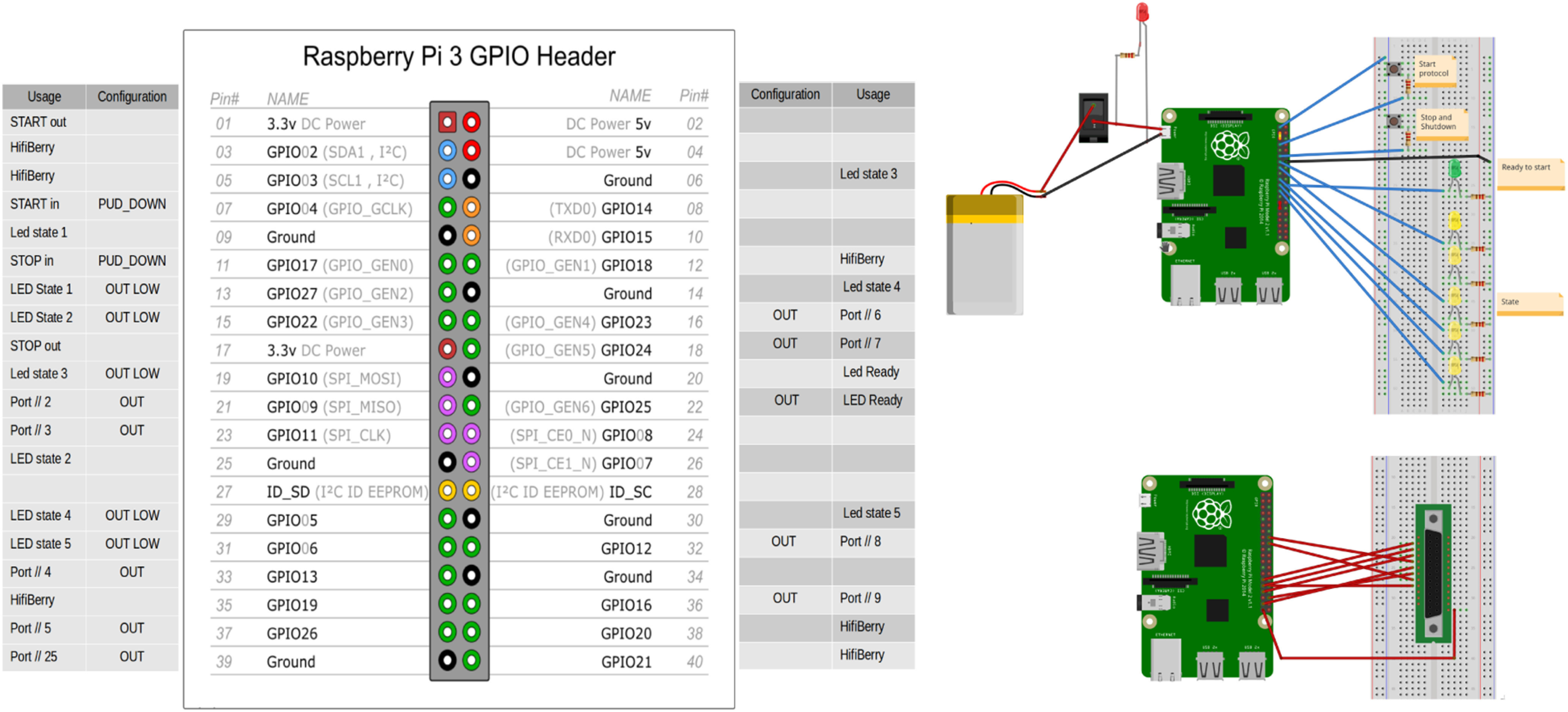
GPIO usage. GPIO pins (3, 5, 35, 38, and 40) are used by the HiFiBerry (not represented in the right diagram); GPIO OUT pins (16, 18, 21, 23, 32, 33, 36, 37, and 39) for parallel port communication (giving 2^8^ = 256 possibilities for markers values); GPIO PUD_DOWN pins (7 and 11) are used for start stop buttons; and GPIO OUT LOW pins (13, 15, 19, 29, and 31) for LED lights indicating the protocol progression to the user.

We used the GPIO to simulate a parallel port as described in Figure 2. It was further used to manually start and stop the systems (using buttons) and to allow visual feedback on protocol progression using LEDs.

**Figure 3.**
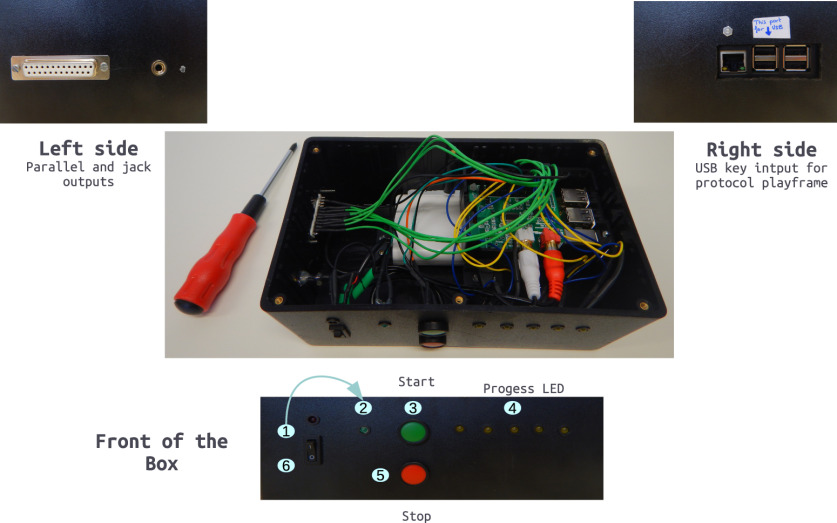
Stimulation box. Top, Output via a parallel port for triggers and auditory jack for sounds (left side), and input via USB keys for the auditory files and playframe (right side). Middle, Top view of the box without its lid. Bottom, Setup procedure (front of the box): (1) switch on the box; (2) wait for the green LED to indicate complete startup; (3) press start to start protocol reading; (4) yellow LEDs indicate progress; (5) end of protocol or urgency stop button; (6) turn box off.

A battery (10,000 milliampere hours; Solo 5, ROMOSS) was used to supply the system with energy while avoiding a connection to a general power outlet. An analog stereo RCA adapter to jack 3.5 female was added for audio output. One USB port was dedicated to a USB key, which allows the deliverance of one playframe and multiple audio files for one participant and one test. Figure 3 shows the finalized box. The overall material cost was <300 €.

### Testing procedure methods

**Figure 4.**
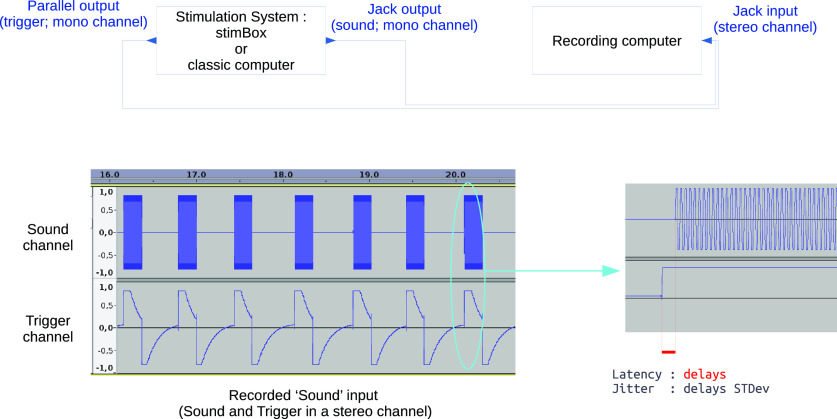
Latency and jitter testing procedure. A recording computer is used to receive and record both outputs of the tested system into one input. One channel is used to record the audio channels as well as the triggers. The difference in onset between the trigger channel and the sound channel are compared. The delay between the two constitutes the latency, while the variance in this latency establishes the jitter (SD).

We measured the latencies and jitters (i.e., latency variability) between audio stimuli and associated triggers for 12 configurations (see below). To realize accurate measurements while avoiding additional timing treatment, we used a direct simple cable physically joining the jack and the parallel outputs from the stimulation system (computer or stimulation box) to a two-paired jack input of a recording computer, as described in Figure 4.

The test (Fig. 4) consisted of playing a 1000 Hz sound a thousand times (sample frequency = 44,100 Hz, duration = 200 ms) coupled with a triggering marker (value = 255). Output from the jack and parallel port were recorded at the exact same moment. A simple threshold (moment where signal is >0) was then used on the recording to detect the exact moment of the sound onset. This was compared with the timing of the trigger occurrence. The difference between each sound onset and the corresponding trigger onset was kept as a latency value, whose distribution across repetitions gives an idea of the jitter (latency standard deviation SD) and its variability.

### Comparison with other systems

To compare these measures with other systems, it is very important to keep in mind that sound performances depend on the wide variety of materials and configurations. First, performance is based on the capacity of the used sound card (e.g., sound fidelity, low jitter performances) and how the sound card interacts with the computer. Indeed, the OS (Windows, Mac, Linux), the selected driver, the software (e.g., Presentation, MATLAB, Opensesame, Psychopy) used and the version used for each one will influence sound control performance.

Moreover, hardware and software interactions are based on an API [application programming interface (also called the “back-end”)] which defines how to manipulate sound data and pilot the sound card. There is a wide range of APIs available within each OS (Windows: MME, DirectSound, WASAPI, ASIO; Linux: ALSA, ALSIHPI, JACK; Mac OS X: Core Audio, JACK). Finally, some parameters could be available for a given hardware/software solution (e.g., sound buffer size, reading mode) that could also have an impact on latency performance.

This exponential variability of possibilities [material * driver * OS * API * software * version * configuration] makes overall benchmark results impossible and shows the necessity of testing a setup before running scientific studies that have strong sound constraints.

**Figure 5.**
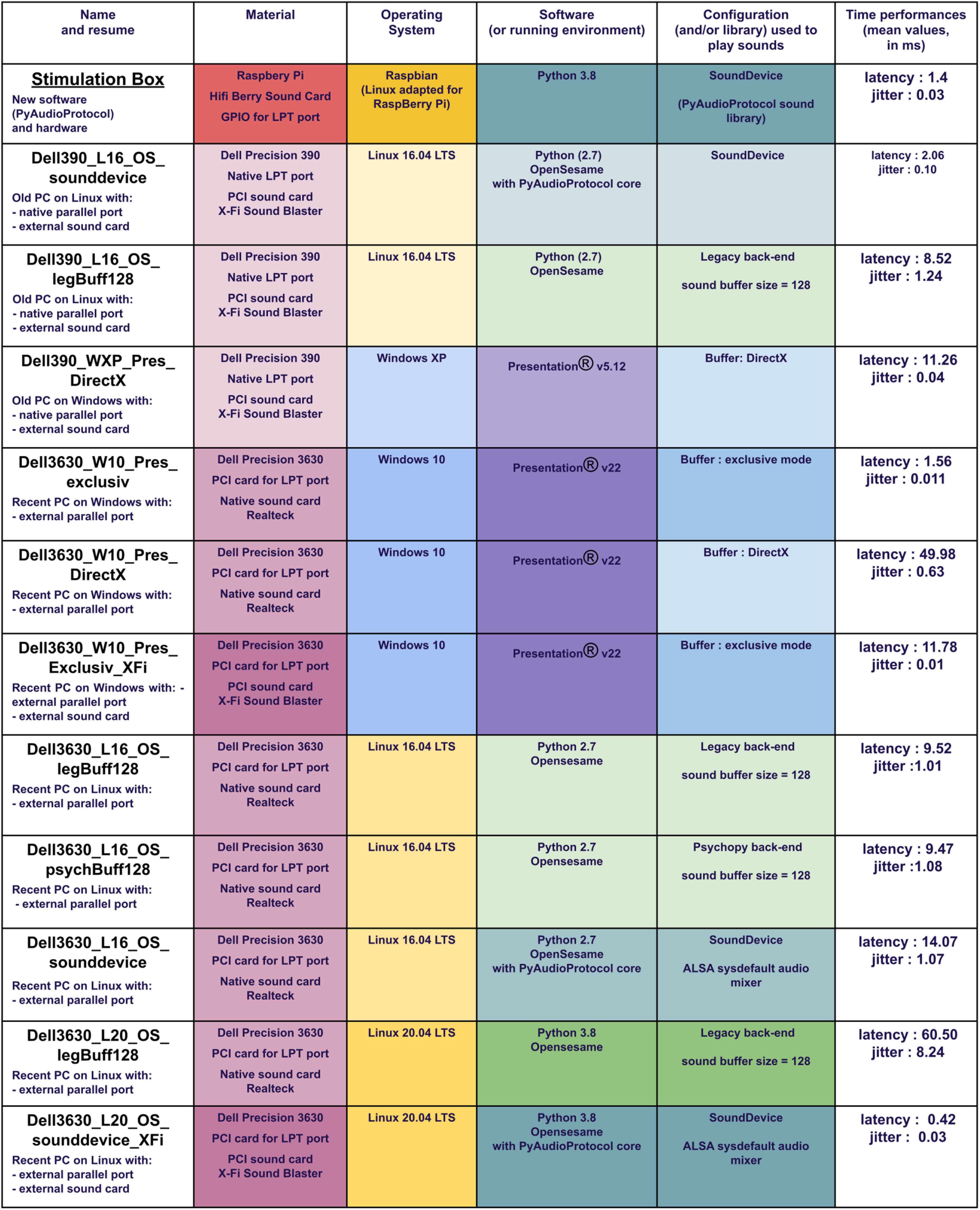
Detailed configurations tested to compare the stimulation box results. It illustrates the latency and jitter variability across different common configurations and variants. Configurations with poor audio quality have been removed.

**Figure 6.**
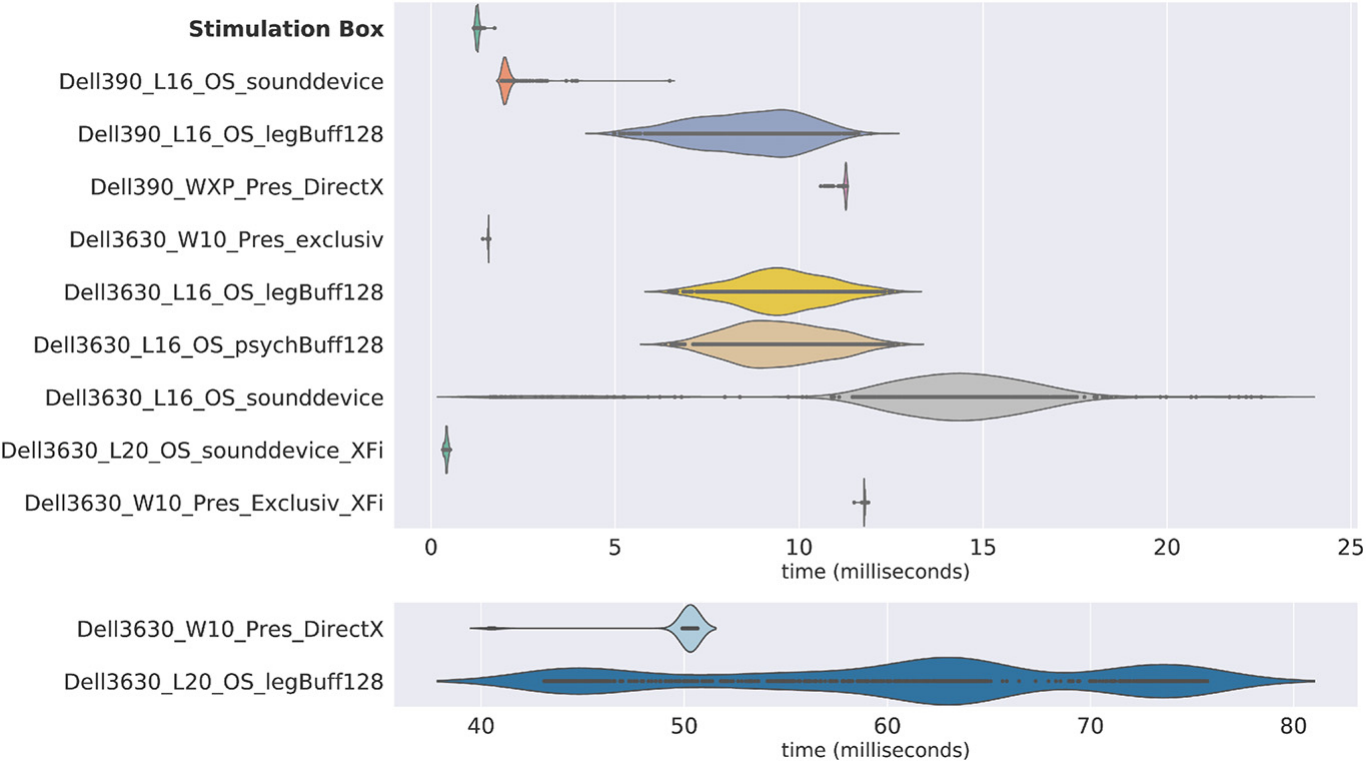
Latencies and jitter results. Results of the stimulation box latency testing are compared with different methods using different computers, operating systems, software, and parameters. Violin plots represent the distribution of the latencies for each of the 1000 sounds and triggers, indicated by points.

For these reasons, we compare our system to some common configurations available to the authors, as detailed in Figure 5. Note that those tests are not covering all the possible solutions but illustrate the variability of the material, OS, software, or version result. Figure 6 in the Results section plots the latency distribution for each configuration, based on the names, as proposed in the first column.

### Data availability

The code described in this article is freely available online at https://github.com/PyOpenProto/PyOpenProto/tree/e-neuro2021/pyaudio_protocol or as the Extended Data 1. 

10.1523/ENEURO.0524-20.2021.ed1 Extended Data 1 playframe_generation_example.py. in Extended_Data1.zip/Project code/example/playframe_generation_example/. Download Extended Data 1, ZIP file.

## Results

Figure 6 shows the latency and jitter test results for the 12 tested configurations. Average latencies range from 0.42 to 60 ms, and average jitter ranges between 0.01 and 8.24 ms in our tested setups. Different solutions showed good latency performance, but the results were not consistent in the case of OS or software version changes. The best tested setups were the Opensesame software running the pyaudioprotocol codes on Linux 20.04 with an external sound card on a recent computer (jitter, 0.42 ms; latency, 0.03 ms) and the stimulation box itself (jitter, 1.4 ms; latency, 0.03 ms). Another good scoring setup was shown to be the Presentation software with exclusive mode on Windows 10 in a recent computer (latency, 1.56 ms; jitter, 0.011 ms).

The same testing protocol was used with longer and variable duration audio stimuli (names and sentences) with very similar results. Further tests with the stimulation box were performed using moving interstimulus intervals. This did not change results either.

### Creation of your own box

All software, as well as a user manual, can be found on GitHub (https://github.com/PyOpenProto/PyOpenProto) under a BSD (Berkeley Software Distribution) license (CNRS CeCill B). A detailed description of the fabrication of a stimulation box can be found on Hackaday (https://hackaday.io/project/181042-stimbox), containing a step by step guide to create the box. A ready-to-use Raspbian operating secure digital card system is available on OSF (https://osf.io/3muqk/). For those who do not wish to create one themselves, you can contact us so that we may redirect you to our collaborators. Furthermore, a 3D version of the stimulation box container for 3D printing is available on Thingiverse (https://www.thingiverse.com/thing:4592271) or as the Extended Data 2.

10.1523/ENEURO.0524-20.2021.ed2 Extended Data 2 Stimulation box container for 3D printing (.stl file and associated pictures). Download Extended Data 2, ZIP file.

## Discussion

This article describes a simple, inexpensive, and open source stimulation system for EEG auditory experiments. It combines hardware such as a Raspberry Pi3 and a HiFiBerry audio card with software for stimulus presentation. The software can be used together with the hardware or as a stand-alone software on a common computer. Both auditory stimulation and trigger timing are optimized when the hardware and software stimulation systems are combined. All information about the Python library, the hardware system, and the configuration settings are open source.

The stimulation box combined with PyAudioProtocol software showed short latency and limited jitters, compared with the 11 other methods. In fact, the results of the stimulation box are among the best for a significantly lower cost. Even if the tests performed were not entirely exhaustive and included only a selection of known configurations used in our research network, they underline the huge variability of possible configuration and associated timing performances. This clearly shows the need for proper specification in research publications and for the standardization of materials through multisite collaborations.

The addition of the HiFiBerry has broadened the range of studies that can be performed using a Raspberry Pi. Previous work from Kuziek et al. (2017) and Mouli et al. (2015) showed that open source stimulation systems could be used to present simple stimulation (beeps). Our solution extends their work by allowing all types of (personalized) audio protocols with good sound quality. In addition, such mobile solutions allow for easy combination with available EEG systems, both classic laboratory-based options as well as the newer open source and mobile EEG options (Pietto et al., 2018; Reiser et al., 2019). Together, this might significantly increase the study possibilities, both in terms of research capacity in smaller laboratories and for experiments outside the laboratory. For example, open-source stimulation and acquisition have already shown their utility in rodents (Mukherjee et al., 2017), and EEG-RaspberryPy2 systems have shown brain–computer interface possibilities (Szewczyk et al., 2020). The open software combined with the accessible and open hardware proposed by our current setup helps to solve some of the challenges that exist concerning standardization for mobile EEG technologies (Lau-Zhu et al., 2019). Furthermore, these new technologies open up a whole field of modular solutions that can simplify and customize studies while being easier to maintain as well as more resilient.

Our system goes back to basics and dissociates the core player from the scheduling intelligence. All necessary ordering of auditory stimulation, from basic to very sophisticated, are prepared in advance in an easy-to-read format (a playframe table, equivalent to a session list file). This limits presentation errors at the study level and facilitates stimulation verification, both before recording and during data analysis. Most importantly, having one USB key per protocol makes experiment setup very quick. Switching from one project and/or participant to another requires only a switch of USB keys and a press of the “start” button.

Currently, the system supports only auditory experiments. Next, improvement will allow the use of a screen to indicate protocol progress instead of the current LED lights. Moreover, a future version will allow the possibility for visual and auditory-visual experiments, as well as adaptive protocols where subject responses are recorded by the system and are used to change the stimulation order. Such improvement will not change the actual audio performances, as they will be handled by different threads or processes. Furthermore, simple adaptations can be made to allow trigger output other than the parallel port. We have also currently added a battery for power to improve mobility and limit connections between the participant and the electrical power lines. However, this could be changed to allow additional external devices. This stimulation system was designed as a shareable project, and we encourage its dissemination, adaptation, use, and further development.

In conclusion, we aimed at creating a user-friendly auditory stimulation system to simplify the creation and implementation of future studies. The portable and easy-to-use stimulation box allows sustainable comparisons between studies and/or centers, which is helpful for reproducibility and collaboration.

Thanks to the development of inexpensive creation tools (e.g., Raspberry Pi, 3D Printing, Arduino), open source languages, like R and Python, and the simultaneous movement toward open science, neuroscience research is able to reclaim transparency, mastery of tools, and excellent practices in our work.

## Synthesis

Reviewing Editor: Macià Buades-Rotger, University of Luebeck Decisions are customarily a result of the Reviewing Editor and the peer reviewers coming together and discussing their recommendations until a consensus is reached. When revisions are invited, a fact-based synthesis statement explaining their decision and outlining what is needed to prepare a revision will be listed below. The following reviewer(s) agreed to reveal their identity: Sarah Tune.

The reviewer and I are satisfied with how the authors have responded to the criticisms and I hence recommend the paper for publication.

## Author Response

1

Synthesis of Reviews

Synthesis Statement for Author (Required):

Dear authors,

Thanks for your work presenting the set-up and performance of an inexpensive, stand-alone

hardware-software combination for auditory experiments. The proposal is timely and in demand; the

solution is feasible and well described. There are however a number of major issues, especially

regarding performance testing (which is unsystematic, lacks a gold standard, and is based on a very

simple paradigm) and the exact intent of the manuscript (as it is neither a tutorial nor a thorough test).

We will consider the manuscript for publication provided you address all issues listed in the

following:

We thank the reviewer(s) for their time and this thorough review with comments that greatly

improved the manuscript. As a summary we feel we were able to respond to the two major

issues. Extra performance testing has been done, and we tried to better explain the strong

system dependency and the impossibility of making such testing systematic. We furthermore

highlight that this is precisely one of the major problems in the field. Of note in the included

new setup is a recent computer and more detailed OS versions and tools. Concerning the intent

of the manuscript, we here show the scientific basis for, and testing off, the presented hardware-software combination as a proof-of-concept. A more detailed tutorial on usage, building process

and associated code will be available on web platforms (GitHub, Open Science Framework,

Hackaday and OpenBehavior; links in the current version of the article have been removed to

ensure anonymity during the eNeuro review process). We however agree that the present article

reads very technical in parts, and less so in others. We have put effort into adapting this

throughout the article.

More detailed comments to the reviewer(s) can be found below.

Major comments

1 - The manuscript, for the most part, is written in a very technical style (e.g. use of terms like

‘protocol specific intelligence’ for something like a session list textfile) and may thus not be very

accessible to a broader audience. In fact, I kept wondering about the exact message and audience of

the manuscript - was it meant as a rather technical benchmark report, or as more of a tutorial to guide

future users through the implementation process? Not all of the potential users of the proposed system

will necessarily have a technical background and would profit from more detailed instructions (more

tutorial-style) on how to use the system. The authors should clearly define the focus, intent, and target

audience of their manuscript and tailor the article -particularly the writing- accordingly.

Response to 1 :

We understand the possible technical feel of the article. We have put effort in clarifying when

possible in less technical terms, but the topic of timing performance is readily strongly technical.

To help the reader being introduced, we added a paragraph in Methods section (2.6 2

‘Comparison with other systems’, page 12 in manuscript version with changes, page 11 for clean

version) about the different hardware and software layers for sound control, that underline the

variability of available solutions. We also inserted a table to improve system comparison

readability. We however feel that one should be able to benefit from and duplicate our work

based on the final version of the article and associated web tutorial links that will be added to

the final version. Indeed, this proof-of-concept manuscript will be combined with open source

guides and how-to manuals after the eNeuro review process. Furthermore, as explained in the

article, we provide a way to order a ready to use version of this box (page 17, line 364 in

manuscript version with changes, page 15, line 317 for clean version).

2 - Regarding the benchmark comparisons. First of all, I’m happy to see the proposed system perform

so comparably well. However, I found the comparisons of different configurations very unsystematic.

Different hardware were paired with different software systems, with the resulting configurations

differing along more than one dimension. This made it very difficult to pinpoint in which respect the

proposed solution performs better than existing systems. E.g. why was the current software (Python

library) tested on an old computer rather than a current one? Having experiment myself a lot with

PsychoPy (also settling on the Sounddevice library in the end) I kept wondering how the proposed

system would compare to my current setup and whether it could improve the timing even more. To

make my point very clear - do the authors wish to imply that the proposed systems works better //

improves upon many currently existing setups (assuming state-of-the-art hardware and current

operating systems) or is the main ‘selling point’ the availability of a comparably cheap and easy to setup system that nevertheless yields overall good performance?

Response to 2 :

Our main selling point is two-fold. Our current system seems to work better than many current

existing setups. Plus, it proposes a cheap and easy to set-up system. To get a clearer

understanding of the different testing setups we have included a summary table to the

manuscript, also added here. With the reviewers comments in mind we included several other

setups and software that are currently available to us in these tests.

The main point is, for this table, to show the huge diversity of setups (depending on tools, OS,

versions, materials, drivers,...) and that a global benchmark comparison of all the cases is not

possible. This is a generally known, but perhaps often overlooked, issue when comparing or

sharing data. It is crucial for experimenters to test the performances of the system used in each

lab if accuracy of stimulation is needed. We hope our setup can help make research more

reliable and collaborations easier.

3 - On a related note, the authors should make clear what is the gold standard for performance and, if

possible, compare their setup to the said standard. This is critical due to the differences in latencies

and, even more critical, in jitter when operating with different computers, operating systems and

software parameters.

Response to 3 :

There is no real ‘gold standard’ for time performance in experiments, but it is mainly defined

by the actual need of associated analysis. If a global analysis is wanted (e.g., FFT over longer

time periods) there is no real need for temporal precision. However, if the goal is to study timelocked performances (e.g., reaction times, ERPs, ERSPs, Phase coherence, etc.) you would like 3

to limit jitter and latencies. The gold standard is then defined by the temporal precision you

need. We briefly touched upon this on page 2 line 43-48 of the manuscript version with changes

(page 2 line 41-46 for clean version). For example, some analyses like phase coherence are very

sensitive to jittering. In the case of evoked potential, it is crucial to get the smallest jitter (i.e

<1ms) whereas the latency could be long (if measured!) as the system latency could be removed

by doing the analysis. In the best case, a short to very short latency (<4ms to <1ms) is

appreciated as analysis correction could be unnecessary. Our system clearly demonstrates such

confident performances.

4 - In its current form, the manuscript refers to a VERY simple auditory presentation paradigm: a

single tone presented along with a trigger. Towards the end of the manuscript the authors allude to

potential extensions to incorporate more sophisticated paradigms. I think this paragraph should be

extended as I think that very few studies would rely on such a simply paradigm only. What about the

presentation of longer auditory stimuli (e.g. longer segments of, say, audiobook) that may need to be

preloaded? What about dichotic presentation of stimuli? How would performance be impacted in such

scenarios? Is accurate timing then only a matter of efficient coding strategies (that handles pre-loading

/ streaming and such things)? What about more realistic scenarios that include visual presentation and

response feedback? How could those additional input // output channels that also need to interface

with the EEG system for triggering be incorporated? The manuscript would be greatly strengthened if

the authors could test a more complex paradigm, else the issue must at least be addressed in the

Discussion.

Response to 4 :

The tests performed in the current manuscript indeed use very simple beeps, because this is the

current standard for testing jitter and latency. Next to defining those basic tests, we tested (and

currently use) the system with longer audio files (words and sentences) and there were no

differences in the result. This is why we decided to keep small waveforms to test accuracy.

As the box pre-loads all audio files, there is no delay or buffer time. Furthermore, the hardware

needs stereo files, especially to create the possibility for dichotic stimuli. Concerning the option

of including visual presentation and response feedback, we agree with the reviewer that this is a

great next step in the development of the software. The hardware explained in the current

manuscript has been created with this in mind, and thus will not need to change. We are

currently in the process of producing this, and are developing a box version with a little screen

as an interface to the user. We strongly believe such a visual representation and response

feedback will not change sound jitter/latency performance, as they could be handled on

different threads. As explained in the manuscript discussion we encourage future users and

collaborators to contribute in order to create an open-source community making this project

successful. However, we feel these efforts are currently out of the scope of the current article.

5 - Please, avoid referencing to clinical research. Authorization of devices for use in human follows

specific rules. This is a device for basic and potentially translational research.

Response to 5 :

Indeed, we are studying the reglementation and testing process to obtain clinical certification,

which is really demanding. The manuscript has been corrected in consequence.4

6 - More as a side note - I was wondering if there is a way within the proposed Raspberry hardware

testing setup (which send the presented audio file directly to the recording computer) to keep

recording the presented audio along with the trigger and EEG. This would be an elegant way to

recover the exact correspondence between EEG and auditory stimulation even in the presence of some

jittering (which could still be detrimental for something like brainstem recordings).

Response to 6 :

We are sadly not entirely sure if we correctly understand the reviewers comment. The

Raspberry hardware testing setup does not record anything, but just sends information to the

recording PC that is used for EEG as well as sending the audio stream to the headphones. The

computer that is used for EEG (and trigger) recording might possibly record the audio track as

well, but this is generally not advised. To do this with the current hardware a simple (stereo)

audio splitter might be used. Instead general advice is to test trigger latency and jitter for

presenting hardware and software before starting a scientific experiment, and adapting the

setup or controlling latency during processing of EEG data.

Simultaneous recording of EEG and audio should not be needed because the

LPT (parallel) port ensures synchronization between devices : the signal, (once sent to the wire)

could be duplicated (as it is an electrical signal) for example for EEG in one wire and audio

recording in another wire. Both sides would receive the signal exactly at the same time (even if

recorded on different machines,..). So it is the synchronization reference : whatever occurs at

the same time than this signal would be synchronized in between. No matter if everything is

recorded on the same computer or not.

But let go through the idea of recording everything on the same computer : the best way to

make it clean would be to record the sound through an EEG electrode. Indeed, EEG data is

recorded by manufactured acquisition softwares that gets data in packets of a defined size. The

trigger is associated to its specific packet with the sample number (included in the packet) when

it occurs. So if we want to record EEG + trig + audio on the same system, outside the EEG

software acquisition, we would need to receive the (EEG+Trig) packet from the acquisition

software (for example from a socket, witch have a latency,..), record the (sound + Trig) in

another way and reading the (EEG and sound) based on the trigger.

Thus only the trigger defines the real synchronization and there is no need to record everything

on the same system if you record the trigger everywhere.

Minor comments

We thank the reviewer for these comments, we have implemented the minor comments and

replied where necessary.

- Please include page numbers.

The manuscript has been corrected.

-The sentence “Such systems do not actually exist” doesn’t read well5

This sentence was removed and the text changed to: “Currently, there is no easy to use,

inexpensive and shareable solution that could improve collaborations and data

comparisons across different sites and contexts. “

-Please, avoid generalizing the tool. It should refer to the particular field of auditory neuroscience. For

example: “ In conclusion, we aimed at creating a user-friendly AUDITORY stimulation systems

that....”

The manuscript has been corrected.

- Please, check spelling. Some usages and wording sounds weird (e.g. improve-upon; heavy technical;

deliverance...)

The manuscript has been corrected.

- Authors should insert a discloser statement due to the many references to commercial equipment

"There are no financial conflicts of interest to disclose.” was included in the ‘disclosure

statement’ line 455 in manuscript version with changes, line 388 for clean version.

- Why refer to the playframe document as ‘excel’ document - it’s simply comma-separated textfile,

right?

Yes, this has been changed.

- With respect to this textfile, the text says “The dissociation of protocol intelligence and playing

process allows the player implementation to focus on general core accuracy in timing and sound

quality while bypassing specific ordering complexity” I know that some presentation softwares also

allow for the order of events and such but as far as I know most available software also allows for

input of externally generated sessionlists.

This is correct and we have added this information in paragraph 2.3

- Fig.3: please add labels to facilitate understanding

We have added labels “right/left/front” to the figure and in the associated text.

- Figure 4 lower panel shows separate audio and trigger channel but caption states that only one

channel is used. Also, “millisecondes” should read “milliseconds”. + latency

The figure has been adapted for clarity and corrected.

- Figure 5: “latence” should read “latency”. I would further suggest increasing the font size for

readability. + ‘milliseconds’

With the new tests, we have adapted Figure 5 completely (and it is now Figure 6).

- Where in the proposed hardware-software setup is the bottleneck that leads to an amount of jittering

that is relatively small but still larger than the best Presentation setup? Could this potentially be

improved upon? Is it due to the intersection of SoundDevice library function with PortAudio?

Response :

Indeed the Presentation setup could have less jittering than the proposed setup. However it is 6

important to keep in mind that this result can only be obtained with one specific presentation

setup (buffer exclusive mode for windows 10), and that it is closed source, payed proprietary

software and only working with Windows. We believe the freely available, open source setup we

propose is already very good. We guess some improvements might be made in the interaction

between PortAudio and the sound card, or the sound card on its own, however this could

similarly improve some of the other PC - back-end options.

- Is there any form of support in place (e.g. via a forum) for potential users to exchange ideas,

download sample codes, collaborate to improve the system even further?

Response :

The code associated with the project will be available on Github platform that allows easy

download, collaborations, version gestion and provides a space for exchanges (‘issues’ for

encountered problems or needs and ‘pull requests’ for collaborations). The project will also be

described on the OpenBehavior and Hackaday websites in the form of a tutorial on how to build

and use the proposed system.

OpenBehavior allows direct communication (via email) and Hackaday provides options for

open discussion through a comment section.

Please note that in the first version of the manuscript, we provided the SD card image used by

the raspberryPi as running environement [Ready to use Raspbian operating system with all

necessary configurations explicited in 2.4 Hardware section]. This file was too big for the new

submission process, so it has not been added as Extended data, but it is available on OSF (Open

Science Framework) website and URL will be added to the final manuscript version (retracted

for double blind review).

- Lines 48-50: The authors write “particularly in research areas in which a temporal accuracy of less

than 10ms, whether as event-related potential or behavioral tests (priming effect), is required.” I

would suggest deleting “(priming effect)", as disambiguating this would require further and

unnecessary elaboration.

The manuscript has been corrected.

- Lines 56-57: the “in Matlab®” at the end might wrongly insinuate that all programs mentioned

before run on Matlab. Please formulate this in a less ambiguous way, e.g. “and Psychtoolbox (Borgo

et al., 2012), the latter in Matlab®.”

The manuscript has been corrected.

- Hardware: please spell out all acronyms (DAC, THD, GPIO and so forth) as they might not be

familiar to the less tech-savvy readers.

Indeed, this has been clarified
