# Supplementary figures and images for: Sharing an Open Stimulation System for Auditory EEG Experiments Using Python, Raspberry Pi, and HifiBerry
Source: eNeuro. 2021 Aug 24;8(4):ENEURO.0524-20.2021. doi: 10.1523/ENEURO.0524-20.2021 (PMC8387158; doi:10.1523/ENEURO.0524-20.2021)

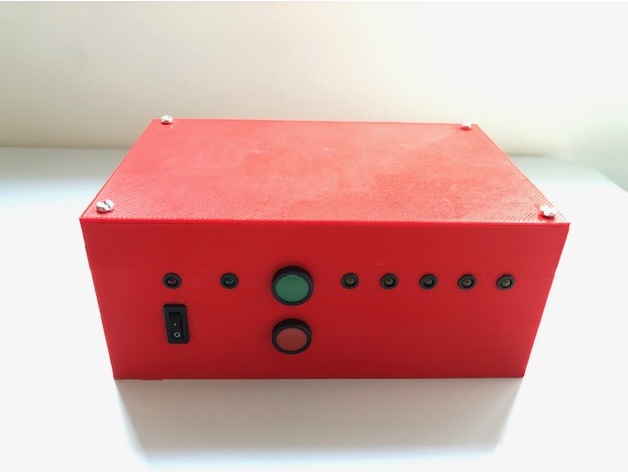

Supplement: Extended Data 2 — Stimulation box container for 3D printing (.stl file and associated pictures). Download Extended Data 2, ZIP file. [file enu-eN-MNT-0524-20-s03.zip › images/IMG_8443.jpg]

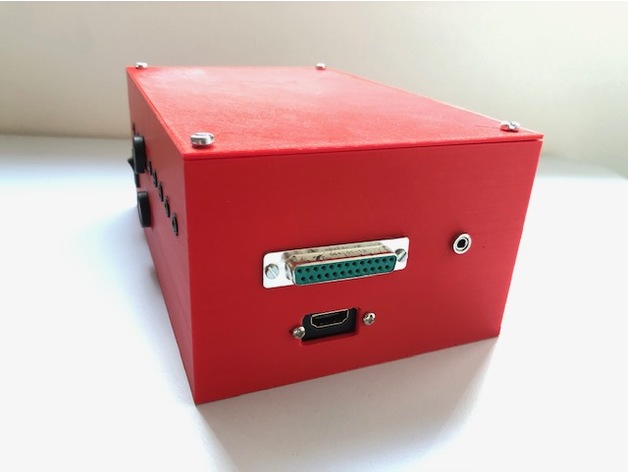

Supplement: Extended Data 2 — Stimulation box container for 3D printing (.stl file and associated pictures). Download Extended Data 2, ZIP file. [file enu-eN-MNT-0524-20-s03.zip › images/IMG_8444.jpg]

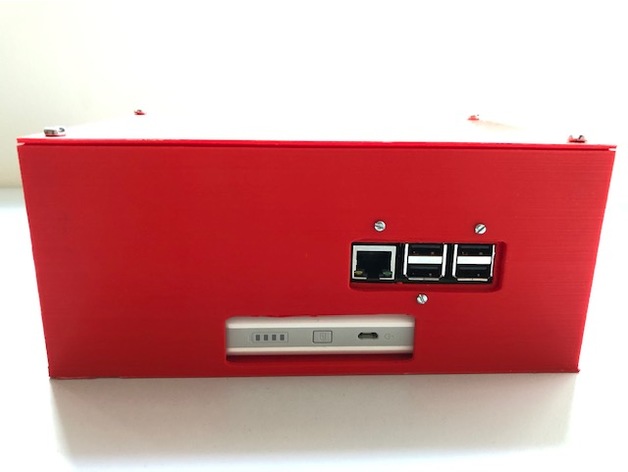

Supplement: Extended Data 2 — Stimulation box container for 3D printing (.stl file and associated pictures). Download Extended Data 2, ZIP file. [file enu-eN-MNT-0524-20-s03.zip › images/IMG_8445.jpg]

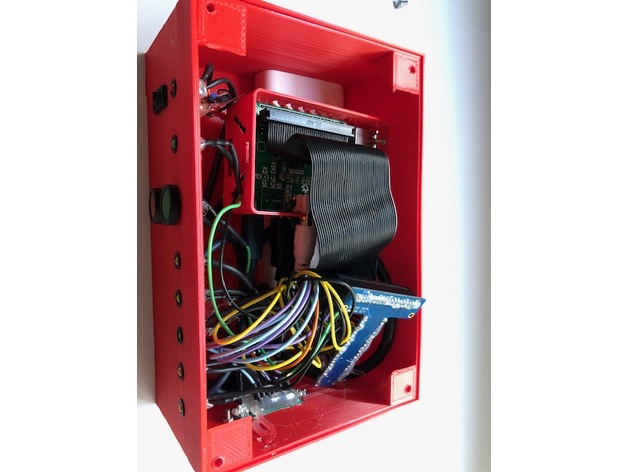

Supplement: Extended Data 2 — Stimulation box container for 3D printing (.stl file and associated pictures). Download Extended Data 2, ZIP file. [file enu-eN-MNT-0524-20-s03.zip › images/IMG_8446.jpg]

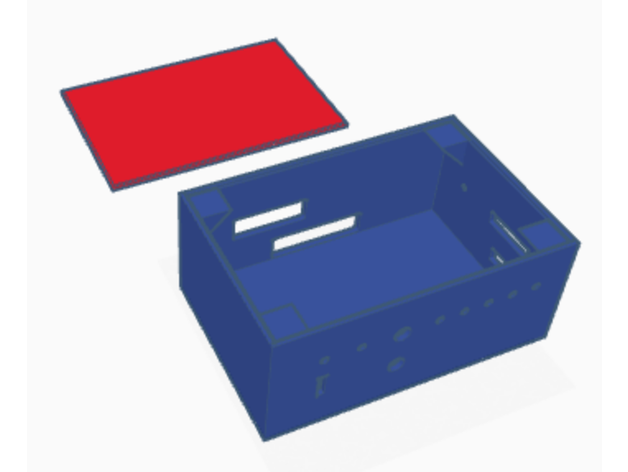

Supplement: Extended Data 2 — Stimulation box container for 3D printing (.stl file and associated pictures). Download Extended Data 2, ZIP file. [file enu-eN-MNT-0524-20-s03.zip › images/stim_box.png]
